# Waist-to-height ratio is a better discriminator of cardiovascular disease than other anthropometric indicators in Kurdish adults

**DOI:** 10.1038/s41598-020-73224-8

**Published:** 2020-10-01

**Authors:** Yahya Pasdar, Shima Moradi, Jalal Moludi, Somaiyeh Saiedi, Mehdi Moradinazar, Behrooz Hamzeh, Mohammad Asghari Jafarabadi, Farid Najafi

**Affiliations:** 1 Research Center for Environmental Determinants of Health (RCEDH), Kermanshah University of Medical Sciences, Kermanshah, Iran; 2 School of Nutritional Sciences and Food Technology, Kermanshah University of Medical Sciences, Kermanshah, Iran; 3 Clinical Research Development Center, Imam Reza Hospital, Kermanshah University of Medical Sciences, Kermanshah, Iran; 4 Nutrition Research Center, Faculty of Nutrition, Tabriz University of Medical Sciences, Tabriz, Iran; 5 Epidemiology, Behavioral Disease Research Center, Kermanshah University of Medical Sciences, Kermanshah, Iran; 6 Department of Statistics and Epidemiology, Faculty of Health, Tabriz University of Medical Sciences, Tabriz, Iran; 7 Social Development and Health Promotion Research Center, Health Institute, Kermanshah University of Medical Sciences, Kermanshah, Iran

**Keywords:** Heart development, Heart development, Nutrition, Nutrition

## Abstract

It has been suggested that abdominal obesity might be a better cardiovascular diseases (CVDs) discriminator than overall obesity. The most appropriate obesity measures for estimating CVD events in Kurdish populations have not been well-recognized. The objective of the present study was, therefore, to determine the cutoff points of BMI, waist circumference (WC), waist-to-hip ratio (WHR), and waist to height ratio (WHtR) as the diagnostic cut-offs to discriminate the prevalent cardiovascular diseases. The data collected from Ravansar Non-Communicable Disease (RaNCD) cohort, the first Kurdish population-based study, was analyzed. The information related to BMI, WC, WHR and WHtR of 10,065 adult participants in the age range of 35–65 was analyzed in this study. Receiver operating characteristic (ROC) analyses were conducted to evaluate the optimum cut-off values and to predict the incidence of cardiac events. The results showed that WHtR had the largest areas under the ROC curve for cardiac events in both male and female participants, and this was followed by WHR, WC, and BMI. The optimal cut-off values for determining the cardiac events in the Kurdish population were BMI = 27.02 kg/m^2^ for men and BMI = 27.60 kg/m^2^ for women, WC = 96.05 cm in men and 99.5 cm for women, WHRs = 0.96 in both sexes, and WHtR = 0.56 for men and 0.65 for women. The current study, therefore, showed that WHtR might serve as a better index of prevalent cardiac event than BMI, WHR and WC.

## Introduction

Obesity, especially central obesity, is defined as fat accumulation around the abdominal area. While obesity is increasing worldwide, it is associated with the risk of cardiovascular diseases (CVDs)^1,2^. Among Iranian adults, 34.8% are overweight and 18.8% are obese^3^. The most common cause of mortality in both developed and developing countries is related to CVDs, imposing many costs on the health care systems^4^. Central obesity has adverse effects on cardio-metabolic indices, including insulin resistance, hypertension and dyslipidemia^5,6^. At present, obesity is identified as a major changeable risk factor^7^.

To identify obesity (i.e., extra body fat accumulation) and the risk of obesity-related complications, several methods such as body mass index (BMI), waist circumference (WC), and waist to hip ratio (WHR) are routinely applied^8,9^. Although BMI has been widely used to screen overweight and obese individuals, this index cannot predict abdominal obesity (i.e., central obesity). Also, it has different age, sex and ethnic-specific standards, making it less practical for parents and non-professional use^5^. Similarly, WC can reflect fat distribution in the abdominal area, but it may vary based on height, sex and race differences^10^. WHR is another simple index; however, this index cannot change with the increase or decrease in both WC or hip circumference^1^. It should be noted that bioelectrical impedance analysis (BIA) is a safe, inexpensive, and non-invasive method for the evaluation of body compositions such as central obesity and body fat accumulation^9,11,12^; however, BIA results are influenced by factors such as ethnicity, environment, phase of the menstrual cycle, dehydration, and underlying medical conditions. So, in large epidemiological studies, more straightforward techniques such as BMI, WC and WHR are regularly used instead of BIA to measure the body fat^13^.

Recently, a new index named waist-height ratio (WHtR) has also gained popularity; it has been used widely for screening the risk of cardiometabolic disorders^14^. This index can provide more accurate information about the obesity status, by considering height, sex and race differences^15^. A recent review has introduced a global boundary value of WHtR < 0.5, which can be helpful in the prevention of CVDs and type 2 diabetes (T2D)^16^.

On the other hand, the most frequently utilized cutoff values of the mentioned indices are mainly based on European and American population data^5,8,17^, which might not be accurately applied in other groups. So, it is necessary to find appropriate cutoff values of each anthropometric index for the early detection and management of CVD risks in other countries and different ethnicity groups^18^.

To the best of our knowledge, there has been no study on the cutoff points of WHtR and other anthropometric indices for the Kurdish population. Therefore, given the obesity rate in Iran, this cross-sectional study was designed to determine the cutoff points of WHtR for the CVDs risk among Kurdish provinces in the West of Iran.

## Methods

### Study design and population

#### Study design

This study was conducted, for the first time, in the Iranian Kurdish cities, or Eastern Kurdistan, an unofficial name representing those parts of the northwestern Iran inhabited by Kurds and surrounded by Iraq and Turkey. This study was carried out using the baseline data obtained from Ravansar Non-Communicable Disease (RaNCD) cohort study. RaNCD has been a population-based ongoing study since 2014, aiming to investigate the non-communicable diseases among 10,065 Kurdish participants (4775 men and 5290 women) in the age range of 35–65 years in Ravansar city, Kermanshah Province, West of Iran. An expert cardiologist screened all of the patients to check the eligibility of conducting the study and pregnant women were excluded because of the high abdominal circumference. An informed consent was obtained from all subjects. This study was part of the Prospective Epidemiological Research Studies in Iran (PERSIAN) mega cohort study approved by the ethics committees at the Ministry of Health and Medical Education, the Digestive Diseases Research Institute, Tehran University of Medical Sciences, Iran. The details of the RaNCD cohort study protocol have been introduced in the previous studies^19,20^. Also, all experiments were performed in accordance with relevant guidelines and regulations. A written informed consent was obtained from each study participant at the beginning of the measurement.

#### Data collection

Data collection and measurements performed by well-trained interviewers in the study site in Ravansar. We used demographic data, anthropometric indices, blood pressure, and biochemical analyses for this study. Participants’ demographic and lifestyle information, including smoking and alcohol consumption history, was collected via face-to-face interviews using a standard questionnaire.

#### Anthropometry

Height was measured by the automatic stadiometer BSM 370 (Biospace Co., Seoul, Korea) with a precision of 0.1 cm while the person was in the standing position without shoes. Weight measurements were performed by InBody 770 device (Inbody Co, Seoul, Korea), we asked all participants to wear the least clothing and without shoes. Height and weight measurement performed only once. BMI was calculated based on the following formula: weight^21^/(Height) (Meter) (meter). WC and HC measured by using non-stretched and flexible tape in standing position three times, and their average was reported. Waist circumference (WC) assessed by measuring the distance around the narrowest area of the waist between the lowest rib and iliac crest and above the umbilicus using a no stretchable tape measure. HC measured at the maximum diameter of the hips. WHR was determined by dividing WC (cm) to HC (cm), also WC (cm) divided by height (cm) was considered as WHtR. The measurement of the anthropometric indices performed by a trained examiner based on the protocol of the RaNCD cohort study.

### Blood pressure

After 4–5 min of rest, Systolic blood pressure (SBP) and diastolic blood pressure (DBP) were measured by conventional sphygmomanometry and auscultation of the Korotkoff sounds in sitting position from both arms of all participants two times. The interval between the two measurements was 10 min and the mean of them was recorded as the final blood pressure^19^.

### Biochemical analysis

After 12 h of fasting, venous blood samples were obtained from all of the participants. Total cholesterol^20^, high-density lipoproteins (HDL), and triglyceride (TG) concentration were measured by enzymatic kits (Pars Azmun, Iran). The Friedewald equation was used to calculate low-density lipoprotein (LDL-C) levels^25^. Fasting blood glucose (FBS) was measured using the hexokinase method^26^.

### Cardiac events

In the current study, we considered the history of CVD, including angina and/or heart failure and/or history of myocardial infarction and/or stroke as cardiac events diagnosed based on participants self-reported and/or medication history examination during a single visit by physicians at the RaNCD cohort health examination center.

### Metabolic syndrome

In our study, the metabolic syndrome (MetS) is defined using the harmonizing metabolic syndrome by the IDF/AHA/NHBLI standard. Any 3 of the 5 following risk factors were considered as a diagnosis indicator of MetS. This criterion included central obesity (waist circumference ≥ 93 cm for Iranian adults) as a national or regional cut point for WC. Other components included: (1) triglycerides ≥ 150 mg/dl, (2) HDL cholesterol ≤ 40 mg/dl for men or 50 mg/dl for women, (3) systolic/diastolic blood pressure ≥ 130/85 mmHg or receiving drug treatment, and (4) fasting plasma glucose ≥ 100 mg/dl^21^.

#### Statistical analysis

All data analyzed by the SPSS (version 18; SPSS Inc., Chicago, IL) and STATA (version 13, Stata Corp, College Station, TX) software, and *P* values < 0.05 were considered as significant. Quantitative data were reported as mean ± standard deviation and quality values were expressed as frequency (%). Pearson correlation coefficient was used to determine the relationship between WHtR with lipid profiles and anthropometric indices. Binary logistic regression in the unadjusted model was used to assess the cardiac events between anthropometric indices and CVD risk factors. By the receiver operating characteristic (ROC) analysis and considering the best combination of sensitivity and specificity, we determined the best cut-off points for anthropometric indices. Likelihood ratios, positive and negative predictive values with 95% confidence intervals were assessed in each cut-off point level for the diagnosis of cardiac events. Besides, the ROC curve comparison tests were used to identify the best anthropometric indices for detecting the best cardiac events predictor. Among semi-parametric, parametric, and non-parametric approaches to estimate the area under the ROC curves, the parametric method appeared the least appropriate for analyzing. Two methods commonly used for establishing the “optimal” cut-point, the point on the ROC curve closest to (0,1) and the Youden index, J. Both have sound intuitive interpretations and are generalizable to weighted sensitivity and specificity hence we used this method^22^. To determine the binary state variable, we use the cardiac events.

## Results

### Descriptive analysis

The general characteristics of the study population were stratified by sex (Table 1). A total of 10,065 participated in the study (5290 women and 4775 men); the mean age was 48.1 ± 8.25 years and the mean o BMI was 26.34 kg/m^2^ in men and 28.56 kg/m^2^ in women. The mean WC was 96.16 cm in men and 98.27 cm in women. In the men group 13.2% were drinkers and 22.5% were current smokers, while just 0.1% and 2% of the participant women were drinkers and current smokers, respectively. Men had higher values of TG, DBP and SBP. There was a marginal difference in LDL (*P* = 0.005), and slight differences were found for other lipid concentrations between men and women.

**Table 1:** General characteristics of the studied population.

Variable	Total (n = 10,065)	Men (n = 4775)	Women (n = 5290)	P*
Age (years)	48.1 ± 8.25	47.82 ± 8.05	48.36 ± 8.41	< 0.001
Weight^21^	72.9 ± 13.64	76.58 ± 13.43	69.6 ± 12.95	0.035
Height (cm)	162.76 ± 9.3	170.33 ± 6.3	155.97 ± 5.56	< 0.001
BMI (kg/m^2^)	27.51 ± 4.63	26.34 ± 4.06	28.56 ± 4.86	< 0.001
WC (cm)	97.27 ± 10.81	96.16 ± 9.99	98.27 ± 11.4	< 0.001
WHR	0.94 ± 0.05	0.94 ± 0.05	0.94 ± 0.06	< 0.001
WHtR	0.59 ± 0.07	0.56 ± 0.05	0.63 ± 0.07	< 0.001
SBP (mm/Hg)	109.67 ± 17.16	111.02 ± 16.78	108.45 ± 17.41	< 0.001
DBP (mm/Hg)	70.66 ± 9.99	71.45 ± 9.86	69.94 ± 10.04	0.155
TG (mg/dl)	137.69 ± 84.32	145.95 ± 89.26	130.25 ± 78.87	< 0.001
HDL (mg/dl)	46.42 ± 11.33	42.8 ± 10.07	49.68 ± 11.42	< 0.001
LDL (mg/dl)	102.08 ± 25.43	101.08 ± 24.65	102.99 ± 26.09	0.005
TC (mg/dl)	185.51 ± 38.02	181.65 ± 36.47	189 ± 39.05	0.001
FBS (mg/dl)	97.06 ± 30.21	97.13 ± 30.98	96.99 ± 29.49	0.942
Diabetic, N(%)	819 (8.19)	378 (7.9)	441 (8.3)	0.499
Metabolic syndrome, N(%)	4194 (41.7)	1837 (38.5)	2357 (44.6)	< 0.001

*BMI* body mass index, *WC* waist circumference, *WHR* waist to hip ratio, *WHtR* waist to height ratio, *SBP* systolic blood pressure, *DBP* diastolic blood pressure, *TG* triglyceride, *HDL* high density lipoprotein, *LDL* low density lipoprotein, *TC* Total cholesterol, *FBS* fasting blood sugar.

*P value was obtained student *T* test and Chi square.

### Discrimination analysis

**Figure 1:**
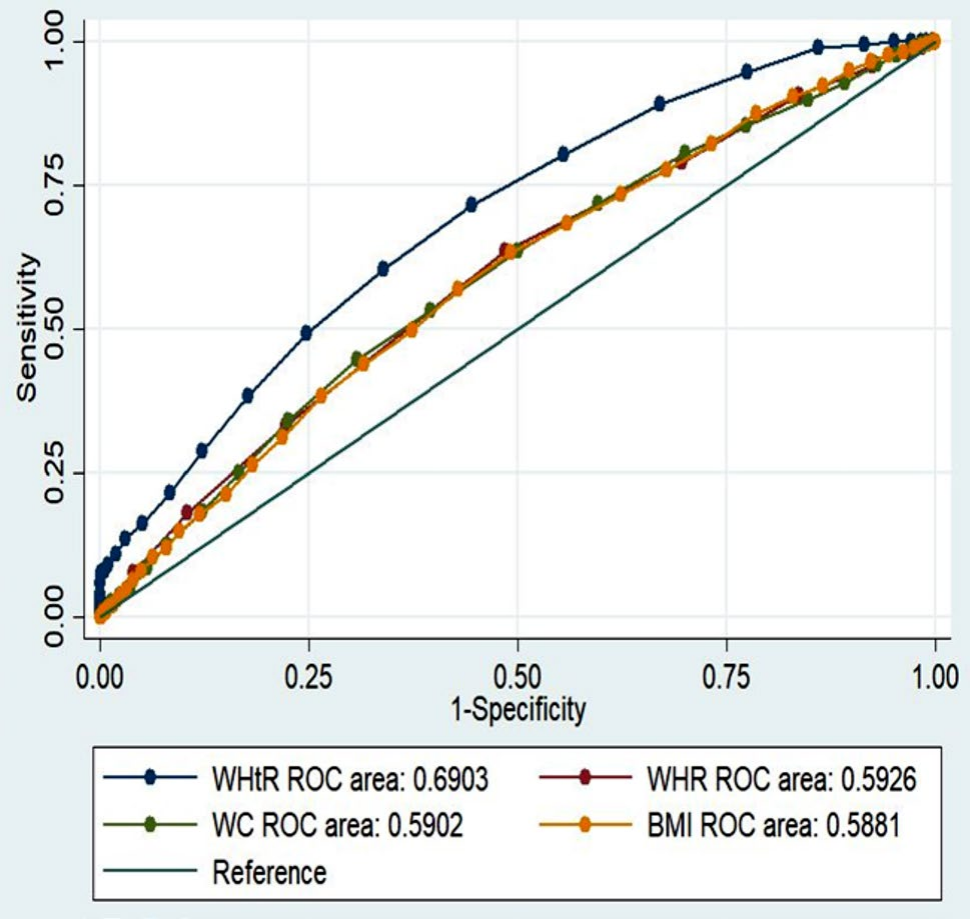
Comparison of AUC of the anthropometric indices for diagnosis of prevalent cardiac event in total.

**Figure 2:**
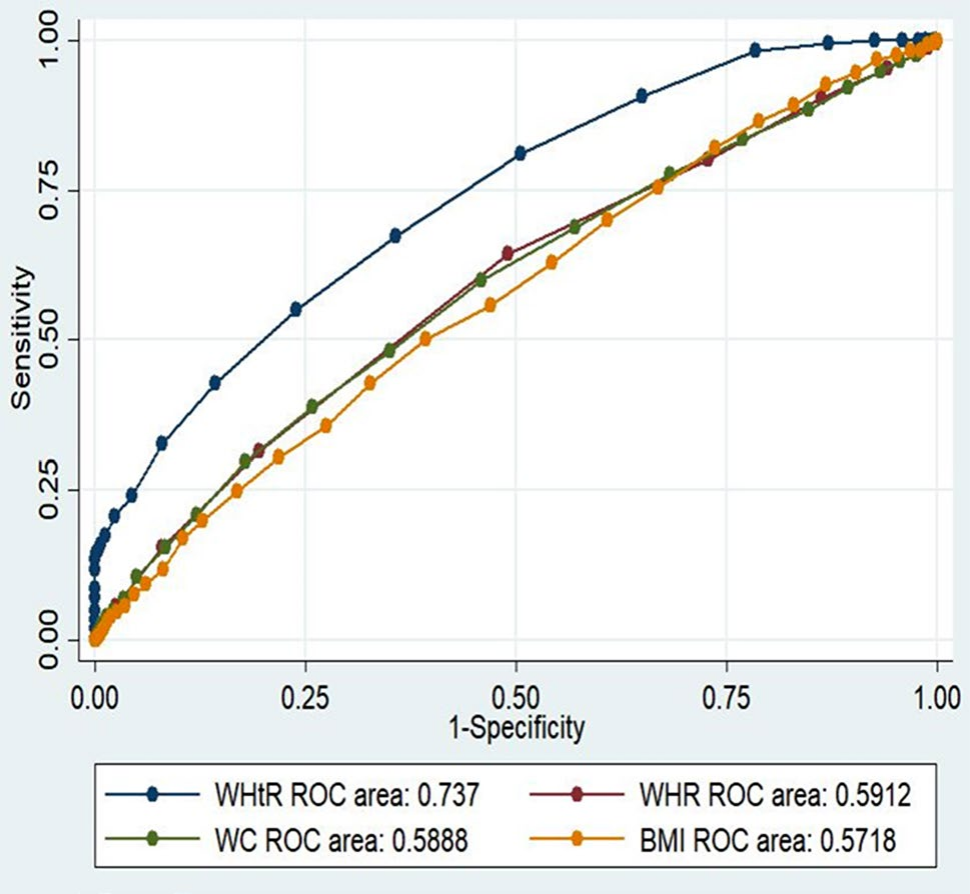
Comparison of AUC of the anthropometric indices for diagnosis of prevalent cardiac event in men.

The area under the curve (AUCs) of the anthropometric indices and cardiac events are shown in Table 2. The AUCs for WHtR were meaningfully higher than those for BMI, WHR and WC. Furthermore, the cut-off values of the various anthropometric indices calculated by ROC analysis for cardiac events are presented in Table 2. The optimal BMI cut-off value for the prediction of obesity-related cardiac events was 27.50 kg/m^2^ in the whole study population. The optimal WC and WHtR cut-off values were 97.15 cm and 0.56, respectively. The area under the ROC curve of WHtR (AUC = 0.69, 95% CI 0.67–0.70) was higher than other anthropometric indices. The AUC for BMI was the lowest among other anthropometric indices AUC = 0.58 (95% CI 0.54–0.59) (Table 2) (Fig. 1). WC showed the highest sensitivity (68%) for prevalent cardiac events, while the sensitivity of BMI (41%) was the lowest. Furthermore, the highest and lowest specificities were revealed for BMI and WC (71%, 43%). The optimal cut-off value of WC in the males was > 96 cm, which was lower than that in the females, i.e. 99 cm; on the other hand, the optimal cut-off for WHtR was > 0.66 in the females, 0.56 in males. Both parameters were found to be better than BMI. The best cut-off value for BMI was > 27.02 kg/m^2^ in the males, while it was > 27.60 kg/m^2^ in the females. WHtR showed the highest likelihood ratios (3.2, 1.4) in men and women, receptively. The LR+ (positive Likelihood Ratio) for WHtR was 3.2 in male, 1.4 in females, subjects were 3.2 (2.6–3.9) and 1.4 (1.3–1.6) times more likely to have cardiac events, respectively (Table 2) (Figs. 1, 2).

**Table 2:** Area under the receiver operating characteristic curve (and 95% confidence interval) and optimal cut-off values of diagnostic measures of anthropometric indices against CVD stratified by sex.

Variables	Men				Women			
	WHtR	WC (cm)	BMI (kg/m^2^)	WHR	WHtR	WC (cm)	BMI (kg/m^2^)	WHR
AUC (95% CI)	0.74 (0.71–0.76)	0.58 (0.56–0.61)	0.57 (0.55–0.59)	0.59 (0.56–0.62)	0.67 (0.64–0.69)	0.57 (0.55–0.59)	0.56 (0.54–0.58)	0.59 (0.57–0.61)
Cut-off	0.56	96.05	27.02	0.96	0.65	99.5	27.60	0.96
Sen (%) (95% CI)	36.6 (30.0–43.6)	28.3 (22.2–35.0)	25.9 (20.0–32.4)	59.5 (52.5–66.3)	59.9 (53.8–65.7)	35.1 (29.5–41.0)	47.3 (41.3–53.4)	50.9 (44.9–56.9)
Spc (%) (95% CI)	88.5 (87.5–89.4)	78.2 (76.9–79.4)	79.1(77.9–80.3)	57.5 (56.1–58.9)	58.3 (56.9–59.6)	68.0 (66.7–69.3)	59.4 (58.0–60.8)	56.1(54.7–57.5)
LR + (95% CI)	3.2 (2.6–3.9)	1.3 (1.0–1.6)	1.2 (0.9–1.5)	1.4(1.2–1.6)	1.4 (1.3–1.6)	1.1(0.9–0.3)	1.2 (1.0–1.3)	1.1(1.0–1.3)
LR-(95% CI)	0.7 (0.7–0.8)	0.9 (0.8–1.0)	0.9 (0.8–1.0)	0.7 (0.6–0.8)	0.7 (0.6–0.8)	0.9 (0.8–1.0)	0.9 (0.7–0.9)	0.8 (0.7–0.9)
PPV(95% CI)	12.5 (9.9–15.4)	5.49 (4.2–7.0)	5.2 (3.9–6.8)	5.9 (4.9–7.0)	7.4 (6.3–8.6)	5.7 (4.7–6.9)	6.1(5.1–7.2)	6.1 (5.1–7.1)
NPV(95% CI)	96.9 (96.3–97.4)	96.1(95.4–96.7)	96.0 (95.3–96.6)	96.9(96.2–97.6)	96.3 (95.6–96.9)	95.0 (94.2–95.6)	95.3 (94.5–96.0)	95.4 (94.5–96.1)
Corrected classified	60	51	36	25	45	52	59	33

*AUC* area under the ROC curve, *Sen* sensitivity, *Spc* specificity, *LR* likelihood ratios, *WC* waist circumference, *BMI* body mass index, *WHR* waist to hip ratio, *WHtR* waist to height ratio.

There are no significant difference among AUCs (*df* = 3) = 130, *P* = < 0.001, (*df* = 3) = 336, *P* =  < 0.001 in men and women respectively. Data are AUC (95% confidence interval).

**Figure 3:**
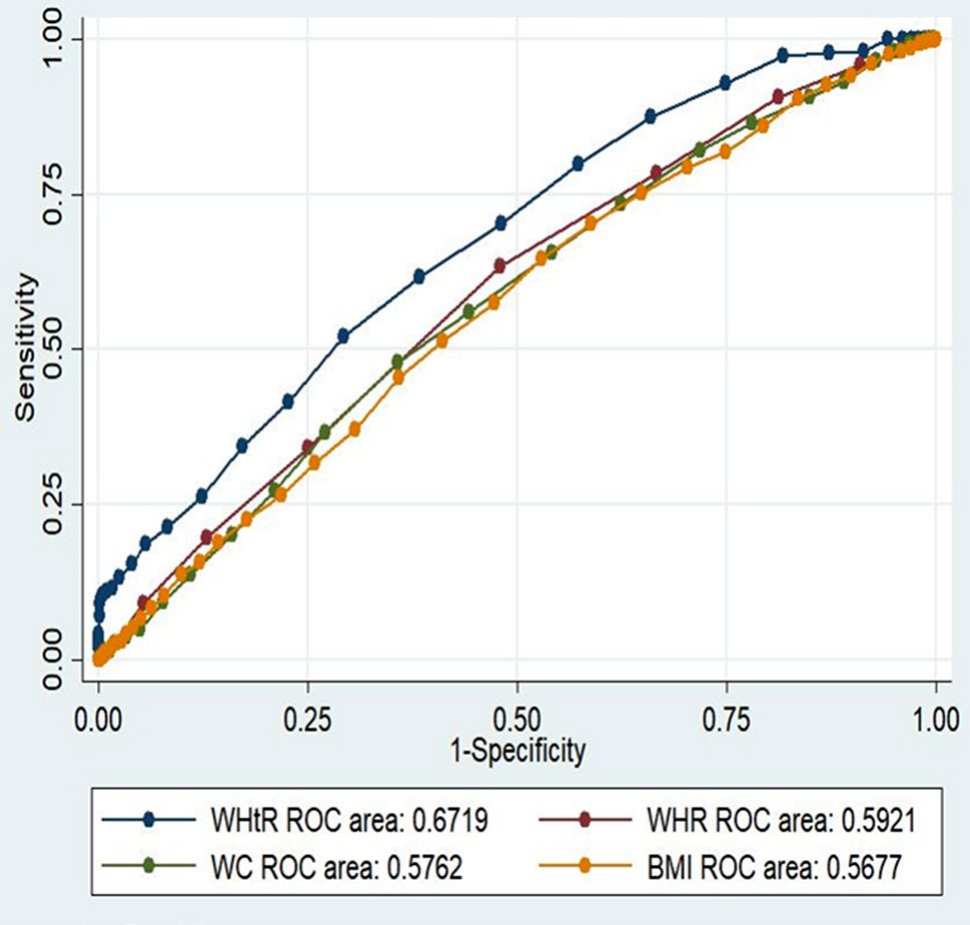
Comparison of AUC of the anthropometric indices for diagnosis of prevalent cardiac event in women.

One analysis assessed the accuracy of the anthropometric index in defining the CVD events. The percentage of individuals classified on the basis of each of the anthropometric indices for the general population was 55% by WHtR, 51% by WC, 49% by BMI and 30% by WHR. In the general population, ROC analysis displayed that WHtR could be a better predictor of CVD events than other anthropometric indices. More importantly, in women, the percentage of individuals classified for WC and BMI was greater than that based on WHtR (Table 2) (Figs. 2, 3).

The AUCs and ROC analysis of the anthropometric indices and MetS can be seen in Table 3. The AUCs for WC were meaningfully higher than those for BMI, WHtR and WHR. The area under the ROC curve of WC (AUC = 0.77, 95% CI 0.76–0.78) was higher than other anthropometric indices for MetS. The optimal cut-off value of WC in the males was > 94.8 cm, which was lower than that in the females, i.e., 98.7 cm; on the other hand, the optimal cut-off for WHtR was > 0.66 in males, and this was 0.56 in females. The optimal BMI cut-off value for MetS prediction was 27.6 kg/m^2^ in the whole study population. The best cut-off value for BMI was > 27 kg/m^2^ in males, while it was > 28.2 kg/m^2^ in the females. The percentage of individuals classified based on each of the anthropometric indices against MetS for the men and women was 71.9% and 68.5, respectively. According to WHtR, it was 70.9 and 57.9%, for WC, the percentages were 61.5 and 58.3% and finally, regarding BMI, 61.5 and 55.7% were obtained by WHR for males and females, respectively.

**Table 3:** Area under the receiver operating characteristic curve (and 95% confidence interval) and optimal cut-off values of diagnostic measures of anthropometric indices against Metabolic syndrome stratified by sex.

Variables	Men				Women			
	WHtR	WC (cm)	BMI (kg/m^2^)	WHR	WHtR	WC (cm)	BMI (kg/m^2^)	WHR
AUC (95% CI)	0.74(0.73–0.75)	0.77(0.76–0.78)	74.1(73.0–75.8)	0.71(0.70–0.72)	0.62 (0.60–0.63)	62.5 (60.0–64.1)	62.3 (60.8–63.8)	0.53 (0.52–0.54)
Cut-off	0.66	94.8	27	0.94	0.56	98.7	28.2	0.95
Sen (%) (95% CI)	73.5 (71.2–74.4)	87.1(85.9–88.3)	62.6 (60.1–64.8)	70.4 (68.4–73.5)	73.4 (69.4–77.5)	57.0 (55.2–59.0)	62.2 (60.1–64.2)	71.8 (70.3–73.5)
Spc (%) (95% CI)	69.2 (68.5–77.6)	61.5 (69.0–52.1)	70.3 (68.5–43.1)	61.3 (58.3–64.3)	65.3 (63.6–67.1)	58.1(56.1–59.1)	56.4 (54.8–58.3)	42.7 (37.3–45.3)
LR + (95% CI)	2.29 (2.17–2.32)	2.3 (2.16–2.39)	2.10 (1.91–2.21)	1.81 (1.41–2.11)	2.0 (1.84–2.17)	1.40 (1.34–1.55)	1.40 (1.21–1.59)	1.2 (1.02–1.41)
LR-(95% CI)	0.5 (0.48–0.55)	0.22 (0.14–0.26)	0.53 (0.49–0.57)	0.48 (0.40–0.52)	0.41 (0.32–0.49)	0.8 (0.72–0.91)	0.70 (0.68–0.72)	0.65 (0.50–0.73)
PPV(95% CI)	60.1(56.0–64.0)	64.1 (61.1–67.0)	63.4 (60.4–66.7)	48.6 (46.9–50.3)	53.7 (51.6–55.8)	53.6 (51.2–56.0)	53.6 (51.5–55.7)	46.2 (44.8–47.6)
NPV(95% CI)	64.7 (63.2–66.1)	68.8 (67.3–70.3)	68.3 (66.7–69.7)	83.9 (82.0–85.8)	62.2 (60.5–64.0)	59.7 (58.1–61.3)	61.7 (59.9–63.4)	83.4 (78.6–87.5)
Corrected classified	71.9	70.9	67.3	61.5	68.2	57.9	58.3	55.7

*AUC* area under the ROC curve, *Sen* sensitivity, *Spc* specificity, *LR* likelihood ratios, *PPV* positive predictive value, NPV negative predictive value, *WC* waist circumference, *BMI* body mass index, *WHR* waist to hip ratio, *WHtR* waist to height ratio.

There is no significant difference among AUCs (*df* = 3) = 130, *P* =  < 0.001, (*df* = 3) = 336, *P* =  < 0.001 in men and women respectively. Data are AUC (95% confidence interval).

## Discussion

This study was intended to compare the anthropometric indices as the predictors of CVD to determine the best diagnostic parameter for cardiac events. Although BMI and WC are widely used, some studies have proposed that WHtR might be a more useful index to assess the adiposity and CVD risk^5,8,16^. The results of the current study indicated that WHtR might be the most accurate measure for detecting CVDs risk, among other anthropometric measurements. The present study, for the first time, revealed the precision, but not the accuracy of using WHtR for defining the cardiac events among Kurdish adults.

Epidemiological evidence shows that obesity could increase the risk of diabetes, CVD, all causing morbidity and mortality^23^. The most serious type of obesity is abdominal obesity, which increases the cardiometabolic risk^24^. There is no specific cutoff point to indicate the individuals at the risk of CVD in different populations^18^. The World Health Organization^25^ has proposed that the BMI cutoffs at which significant cardiometabolic risk is developed varies from 26.0 to 31.0 kg/m^2^, depending on the country, and there has been no prior study specifying the cutoff points for the Asian population, especially in the Iranian subjects with different ethnic or cultural backgrounds^25^. Previous studies have recommended that different BMI cut‐off points must be renewed among race/ethnic populations for better sensitivity and specificity^26^. The results of the present ROC analysis indicated that the BMI cut-off value for diagnosing cardiac events in men was 27.02 kg/m^2^ and 27.60 kg/m^2^ in women. Our results are in line with a longitudinal study conducted in the Iranian population to determine the cutoff values of the anthropometric variables in order to predict CVDs. Hadaegh et al.^27^ highlighted that the BMI cut-off point to predict various CVD risk factors in the Iranian population was 29.2 and 29.5 in men and women, respectively. Also, in other studies, it has been shown that the BMI cut-off point considered to predict various CVD risk factors in the Asian population was 23 kg/m^2^ in men and women^18,28^. The cut-off values obtained by our study for the Iranians were higher than those suggested for Asians and Caucasians: a finding consistent with several^28,29^ but not all previous studies.

WC ignores the effect of height on the cardiometabolic risk; so, some studies have highlighted that WC might be a better indicator of CVD risk than BMI in many race-ethnicity groups^30,31^ and abdominal fat accumulation; the high WC has been proved as a more reliable predictor of the risk of CVD in comparison with the overall obesity. In the western population, the WC cut-off value for defining the abdominal obesity is 102 cm for males and 88 cm for females^32^. There are still no clear WC cutpoints for the western Asians population^33,34^. Our results suggest that a WC of 96.05 cm in men and 99.5 cm in women should be considered as the cut-off points for CVD events prediction in the Kurdish adults. However, due to the low accuracy of this cut-point, it is not currently recommended. These results, therefore, indicated the inconsistency in WC thresholds in terms of sex, race and even country of origin^29^.

On the other hand, the specific cut-off point of a particular country cannot be generalized to all Asian Populations. The optimal WC cutoffs for predicting the cardiometabolic risk may differ among various Asian countries^33^; it is highly recommended that optimal WC cutoffs for abdominal obesity be attained across sexs or races. More recently, in a nationwide study of Iranian subjects, the means of WCs among women and men were 91 and 89 cm, respectively^35^. A higher WC in the Iranian women, as compared to other nations, might be attributed to their inactive life styles, genetic features, low smoking and high fertility rates^36^.

In line with the previous studies, as stated above, WC and BMI have some limitations in predicting the cardiovascular risk factors. It has been proposed that WHtR may be a simple anthropometric index for estimating the cardiometabolic risks in comparison to other anthropometric indices^37^. The results of our ROC analysis demonstrated that in both males and females, WHtR could be a better predictor of CVD events than WC, WHR and BMI; on the other hand, in another study, in the Australian population, it was found that WHtR was not better than WC or BMI in forecasting the CVD mortality^38^. In our study, the WHtR cut-off values considered to identify CVD events were 0.56 in men and 0.65 in women. These results were in close agreement with the findings of the previous studies carried out in the Iranian population^27,39^. Furthermore, the cut-off value of 0.5 for WHtR has been proposed as a better predictor of the CVD risk for both sexes in the European populations^40^. This is in agreement with our results, demonstrating that WHtR ≥ 0.5 is usually more sensitive (based on the ROC analysis) than WC ≥ 96 cm in women, and WC ≥ 96 cm in men, for the screening and diagnose of the CVD events^8,15^. Therefore, WHtR may have better efficacy than other anthropometric indices as a quantity of obesity to screen for CVD risk factors. WC has appeared as the second best anthropometric indicator for the screening of the CVD events. The greater AUC for WHtR in different population groups supports its use as a reliable screening tool for adiposity. Furthermore, the WHtR index overcomes some of the limitations of WC as it is adjusted for height. This is because, against other indices of abdominal adiposity, a world-wide cut-off points has been recommended for WHtR in numerous studies, apart from the necessity for age-, sex- and ethnic-specific cut-off standards^1,14,37^. While WHtR may be superior to BMI and WC in its discriminating power for the CVD events, as compared with WC, WHtR improved the discrimination of CVD by 9%. However, due to poor corrected classification, all of the indices in our study did not perform well enough to diagnose the CVD events. A value of 100% would suggest perfect discrimination, whereas 50% might show discrimination no superior than chance. In fact, most anthropometric measures are only expected to evaluate the cardiovascular risk factors with 60–70% accuracy^8,14,15,17,32^, as observed in our study; therefore, they should be regarded as the first‐stage, or population‐based, screening measures.

It seems that the effects of WC and BMI might be mediated by insulin resistance and other mechanisms such as inflammatory response. On the other hand, the application of WC to evaluate the CVD risk factors also lends support to the idea, although mistakenly, that risk stratification is not susceptible to individual height. The results of our ROC analyses demonstrated that WHtR was better than BMI and WC in predicting the CVD events in both sexes; interestingly, it was the best predictor in men. However, in women, the percentage of individuals correctly classified based on BMI and WC was greater based on WHtR. This could be related to the fact that BMI and WC might reflect the total body fat better than WHtR in women^41^. Furthermore, we examined anthropometric indices with cardiac events alone and with drugs-related CDV. All of the above were calculated as cardiac events and a sensitivity analysis excluding those participants having drugs-related CVD was conducted; the results did not change significantly.

Moreover, our results showed that women in the current survey were of a shorter height, as compared to European and Caucasian populations, which would cause the higher amount of body fat, as compared to the controls of the same ethnic group^42,43^. This evidence might possibly explain why WHtR and BMI are less sensitive tools for identifying CVD events in the short subjects.

Our results study showed that WHtR, as well as BMI and WC, could be useful for predicting MetS, which was consist with the study conducted by Rajput et al.^44^ Based on our AUC analysis, BMI, WC and WHtR were predictive of high metabolic risks in males (0.740, 0.770, and 0.740, respectively), whereas WHtR, BMI and WC were equally predictive of high MetS risks (0.62) in females. Liu et al.^45^ in their study of Chinese population, based on the ROC analyses of BMI, WC and WHtR values, indicated that the presence of multiple metabolic risk factors could be equally predicted, and the AUC values of BMI, WC and WHtR did not differ in men and women (0.702, 0.671 and 0.674, respectively). The appropriate cut-off values for BMI, WC and WHtR were 22.9 and 23.3 kg/m^2^, 91.3 cm and 87.1 cm, and 0.51 and 0.53 in men and women, respectively. Zeng et al. revealed that the ideal cut-off values to define obesity in Chinese adults were nearly 24.0 and 23.0 kg/m^2^ for BMI, 85.0 and 75.0 cm for WC, and 0.50 and 0.48 for WHtR in men and women, respectively. Ashwell et al. also stated that WC improved the identification of harmful CVD risk outcomes by 3%, as compared with BMI, and WHtR, enhancing the diagnosis by 4 to 5%. Additionally, WHtR was a robust prognosticator in comparison to WC CVD and^37^. In our cohort study, the best WHtR cutoff values for predicting MetS, according to WHtR, were 0.66 for men and 0.56 for women. The prevalence of MetS would be different based on various anthropometric indices^46,47^ and the criteria of MetS require more investigation to get a reliable and specific marker in order to classify MetS. Since obesity is the primary cause of the metabolic syndrome, it is essential to use the most reliable index to diagnose it. Furthermore, WHtR is reported as a simple and favorable index to identify MetS; some studies have also shown that the WHtR of more than 0.5 as the cut-off point can recognize people with a higher risk of MetS^47^. On the other hand, WHtR provides a universal cutoff value equally applicable in both sexes and different ages^48^. Such advantages have been summarized in the following public health motto: “Keep your waist circumference to less than half of your height”^40^.

The notable strength of the current study was the availability of the comprehensive data related to more than 10.000 subjects. However, there are some limitations; first, the data concerning diet and physical activity were not taken into account in the analysis because of the scarcity of such information. Secondly, we had to use the CVD events as the dependent variables to test WHtR and it was necessary to extend these findings, which were stratified by cardiovascular disease risk factors. The other limitation is that anthropometric measurements were measured once, while two measurements are usually needed; in some cases, three repeats are even required. Furthermore, it must be emphasized that for the cut-off point drawn from this study, based on the study of Kurdish people in the western part of Iran, additional studies are still needed to conclude that our results could be applied to other populations. Additionally, the lack of body composition analysis and skinfold thickness values to measure adiposity was another limitation in our study.

## Conclusion

WHtR is a simple and effective index to predict the presence of the CVD events in the Kurdish population; so, it might be a better predictor than WC and BMI. Indeed, we could conclude that a WHtR of > 0.56 is better for the diagnosis of a population with CVD events. Our analyses highlighted the variability of the optimal cutoff points for different anthropometric indices among men and women; it is necessary to organize them to determine the optimum cut points in different Asian countries, especially in the Iranian women. Further studies are also required to determine the precise cut-off points to extend these findings for cardiovascular disease risk factors. Finally, we recommend that BMI = 27.02 kg/m^2^ in men and 27.60 kg/m^2^ in women, WC of 96.05 cm in men and 99.5 cm in women, WHRs of 0.96 in both, and WHtR of 0.56 in men and 0.65 in women would define the CVD events in the Kurdish population. Furthermore, the present study showed the precision but not the accuracy of using WHtR for defining the cardiac events among Kurdish adults for the first time. So, these anthropometric indices may be used as a noninvasive approach that could be useful as a prescreening tool rather than a diagnostic one.
